# Agreement between parent reported and clinical coding of asthma, eczema and allergic rhinitis: The multi‐ethnic Born in Bradford cohort

**DOI:** 10.1111/pai.70166

**Published:** 2025-08-07

**Authors:** Gillian Santorelli, Lucy Pembrey, John Wright

**Affiliations:** ^1^ Bradford Institute for Health Research Bradford Teaching Hospitals NHS Foundation Trust Bradford UK; ^2^ Department of Medical Statistics London School of Hygiene & Tropical Medicine London UK

**Keywords:** childhood allergic diseases, childhood asthma, diagnostic agreement, electronic health records, parent report, predictive validity

## Abstract

**Background:**

Discrepancies between parent reports and electronic health records (EHRs) challenge the accurate estimation of childhood allergic disease prevalence. This study aimed to: (1) compare parent reports of asthma, eczema, and allergic rhinitis with GP‐recorded diagnoses; (2) identify factors associated with reporting differences; and (3) assess the predictive validity of parent reports for future diagnoses.

**Methods:**

Data were analyzed from 2594 children (aged 4–5 years) in the UK Born in Bradford (BiB) cohort. Parent‐reported symptoms and diagnoses from questionnaires were compared against diagnoses in primary care EHRs. Agreement was assessed using prevalence estimates and agreement metrics. Logistic and Poisson regression models were used to identify factors influencing reporting and to evaluate predictive validity.

**Results:**

Agreement varied by condition. For parent‐reported “ever‐diagnosed” asthma, agreement with GP records was good (Kappa = 0.68), while for recent eczema symptoms, it was poor (Kappa = 0.07), though this improved after adjusting for prevalence (PABAK = 0.66). Parent reports were highly reliable for ruling out diagnoses. Factors including ethnicity and GP visit frequency were associated with reporting discrepancies. Parent reports at age 4–5 strongly predicted a future GP diagnosis, increasing the risk fivefold for asthma and threefold for allergic rhinitis.

**Conclusion:**

Neither parent reports nor EHRs alone capture the full picture of childhood allergic disease. Parent reports offer crucial insights into symptom burden and future risk, while EHRs provide objective diagnostic data. An integrating approach, combining both sources, is essential for comprehensive epidemiological research and a more complete understanding of disease burden.

**Key message**

In a large, multi‐ethnic UK study, this study found significant and nuanced discrepancies between parent‐reported allergic conditions (asthma, eczema, and allergic rhinitis) and diagnoses in primary care electronic health records (EHRs). While overall statistical agreement was often poor, particularly for allergic rhinitis, parent reports demonstrated two key strengths: they were highly reliable for ruling out a GP diagnosis, and reports at ages 4–5 strongly predicted a formal diagnosis in later childhood. Discrepancies arise from several factors, including mismatched timeframes, differing parent and clinician thresholds for what constitutes a case, and the fact that not all parent‐observed symptoms—especially milder ones—result in a formal GP consultation. The findings demonstrate that neither source alone provides a complete picture. Therefore, for a comprehensive understanding of disease burden and to avoid deepening health inequalities, future research and practice must integrate the essential parental voice with the objective, longitudinal data from EHRs.

## 1 BACKGROUND

Asthma, atopic eczema, and allergic rhinitis are common childhood conditions that reduce quality of life^1^ and increase the healthcare burden.^2^, ^3^, ^4^ In the UK, prevalence estimates among children are approximately 9% for asthma,^5^ 20% for atopic dermatitis,^3^ and 10%–15% for allergic rhinitis.^3^ These conditions often coexist, partly due to shared genetic susceptibilities.^6^

Disease prevalence is estimated using diverse data sources, including questionnaires, clinical assessments, and medical records. Parent‐reported survey data are common in epidemiology due to their efficiency, but they are prone to interpretation and recall bias. The landscape of health data is shifting; the increasing use of electronic health records (EHRs) offers a valuable alternative to costly surveys, providing direct access to clinical diagnoses for disease surveillance and research validation.

Assessing the concordance between parent reports and EHR diagnoses is essential for interpreting disease estimates and selecting reliable data sources. Previous research comparing parent‐reported asthma diagnoses with primary care records shows mixed findings. Some studies found moderate^7^ or good^8^ concordance for doctor‐diagnosed asthma, while others observed more parent‐reported wheezing symptoms than documented diagnoses, with agreement improving as children age.^9^ Similar discrepancies exist for atopic dermatitis,^10^, ^11^ but less is known about allergic rhinitis. The factors driving these discrepancies are largely unknown.

Parent‐reported symptoms without a corresponding GP diagnosis may represent a significant, unrecognized disease burden, potentially delaying care. While concurrent agreement has been studied, the extent to which early parent‐reported symptoms predict future clinical diagnosis remains largely unexplored.

This study aimed to (1) compare parent‐reported symptoms and diagnoses of wheeze/asthma, eczema, and allergic rhinitis with GP‐recorded diagnoses; (2) identify factors associated with parental under‐ and overreporting; and (3) assess the predictive capacity of parent reports for future diagnoses.

## 2 METHODS

### 2.1 Setting and participants

The study used data from the Born in Bradford (BiB) cohort, a large, multi‐ethnic health research project that enrolled 12,453 pregnant women in Bradford, UK, between 2007 and 2011, resulting in 13,858 births.^12^, ^13^ This analysis used participants from the BiB‐MeDALL sub‐study, recruited between October 2012 and June 2015 to investigate IgE‐associated allergic diseases.^14^ Eligible BiB children (born from September 2008 with available maternal and cord blood samples; *n* = 4689) were invited; 2594 (55.3%) participated. At recruitment, children were aged 4–5 years. Parents/carers consented to routine data linkage, with ethics approval from Bradford Research Ethics Committee (Ref 07/H1302/112).

### 2.2 Parent‐reported outcomes

Parents completed the harmonized MeDALL questionnaire about symptoms and diagnoses of wheeze, asthma, atopic eczema, and allergic rhinitis.^15^

Wheeze within the past 12 months was defined as a positive response to the question ‘Has your child had wheezing or whistling in the chest in the past 12 months?’. Severe wheeze in the past 12 months was defined as a positive response plus one of the following: ≥4 attacks of wheezing, sleep disturbances ≥1 night/week, or wheeze limiting speech. Parent‐reported GP‐diagnosed asthma was a positive response to ‘Has your child ever been diagnosed by a doctor as having asthma?’

Atopic eczema in the past 12 months required a positive response to ‘Has your child had an itchy rash which was intermittently coming and going at any time in the past 12 months?’ and the rash affecting classic locations (e.g., elbows folds, behind the knees). Severe eczema included these symptoms plus sleep disturbances ≥1 night/week. Parent‐reported GP‐diagnosed eczema was a positive response to ‘Has your child ever been diagnosed by a doctor with having eczema/atopic dermatitis?’

Allergic rhinitis in the past 12 months was a positive response to ‘…has your child had a problem with sneezing or a runny or blocked nose when (he or she) did not have a cold or the flu?’. Severe cases were not analyzed due to low numbers. Parent‐reported GP‐diagnosis of allergic rhinitis was a positive answer to ‘Has your child even been diagnosed by a doctor with having allergic rhinitis or hay fever?’

### 2.3 GP‐recorded diagnoses

Diagnoses were identified from primary care EHRs via the SystmOne software (© TPP), which is used by all GP practices in Bradford. A 98% match rate was successfully obtained between study participants and their EHRs. To identify cases, we used specific codes from CTV3 (Clinical Terms Version 3), a standardized clinical thesaurus historically used across the National Health Service (NHS). Following the broad case definitions established in our previous work,^16^ a child was defined as having a condition if their record contained at least one relevant CTV3 code for asthma at age three or older, eczema at age one or older, or allergic rhinitis at any age. These minimum age thresholds were set to avoid the known difficulties in differential diagnosis in infants. If a child's record contained none of the codes meeting these definitions, the condition was considered absent.

### 2.4 Covariables

Maternity records provided mother's age, parity, and child's sex. Maternal ethnicity, smoking status, and education were obtained from the baseline questionnaire. Maternal BMI was calculated using height measured at recruitment and weight from pregnancy booking. Parental history of asthma, eczema, and allergic rhinitis was identified using the same method applied for the children. The number of each child's GP attendances in the year before questionnaire completion was sourced from primary care records.

### 2.5 Statistical analysis

#### 2.5.1 Agreement analysis

We conducted a cross‐sectional analysis to evaluate the agreement between health conditions reported by parents and GP‐recorded diagnoses. To ensure temporal alignment, GP diagnoses were censored at the date of questionnaire completion; any diagnosis recorded after this point was excluded from the case definition. We calculated prevalence from both sources, the absolute prevalence difference, overall agreement, Cohen's Kappa (poor < 0.20, fair 0.21–0.40, moderate 0.41–0.60, good 0.61–0.80, very good 0.81–1.00),^17^ and the Prevalence‐Adjusted Bias‐Adjusted Kappa statistic (PABAK) to correct for the ‘Kappa paradox’ in low‐prevalence conditions.^18^ Using the GP diagnosis as the gold standard, we also calculated sensitivity, specificity, positive predictive value (PPV), and negative predictive value (NPV).

Two timeframes were used for comparison. For 12‐month symptoms, we compared parent answers to GP diagnoses made within that same 1‐year period. For “ever” diagnoses, we compared parent answers to the child's cumulative GP records (applying the minimum age thresholds). A secondary analysis compared the 12‐month symptom reports against the cumulative GP records, applying the same age criteria.

#### 2.5.2 Analysis of influencing factors

We identified characteristics associated with parental underreporting (not reporting a confirmed GP diagnosis) and overreporting (reporting a non‐existent GP diagnosis) using unadjusted and fully adjusted logistic regression models on imputed data, with robust standard errors to account for clustering by mother. For this analysis, GP diagnoses made after the questionnaire completion date were not considered cases.

#### 2.5.3 Predictive validity analysis

We evaluated whether parent reports at 4–5 years predicted a new GP‐recorded diagnosis within the subsequent 5 years. Children with an existing GP diagnosis were excluded. Poisson regression (on imputed data, with methods mirroring the above analysis) estimated relative risks (RR) of future diagnosis.

#### 2.5.4 Missing data

We used multiple imputation to generate 20 new datasets to address missing data for maternal BMI, smoking, educational attainment, and parity.

## 3 RESULTS

### 3.1 Participant characteristics

Table 1 presents the study population's characteristics. Most mothers were of South Asian ancestry (62.8%) and multiparous (64.3%). The mean child age at questionnaire completion was 4.0 years (SD 0.2).

**TABLE 1:** Characteristics of study sample.

Characteristic	*N* = 2594
Mothers	
Age at delivery (years)	28.6 (5.6)
Ethnic group	
White European	808 (31.2%)
South Asian	1629 (62.8%)
Other ethnic group	157 (6.1%)
Missing	0
BMI	
Healthy weight	1291 (49.9%)
Overweight	765 (29.6%)
Obese	532 (20.6%)
Missing	6
Smoked during pregnancy	
No	2337 (90.3%)
Yes	252 (9.7%)
Missing	5
Educational attainment	
Less than degree level	1866 (72.1%)
Degree level	722 (27.9%)
Missing	6
Parity	
Nulliparous	899 (35.7%)
Multiparous	1620 (64.3%)
Missing	75
GP diagnosis of asthma	462 (17.8%)
GP diagnosis of atopic eczema	770 (29.7%)
GP diagnosis of allergic rhinitis	675 (26.0%)
Children	
Age at questionnaire completion (years)	4.0 (0.2)
Sex	
Male	1301 (50.2%)
Female	1293 (49.8%)
Number of GP attendances^a^	
0	313 (12.1%)
1–5	1603 (61.8%)
6–10	516 (19.9)
>10	162 (6.3)
GP diagnosis of asthma	449 (17.3%)
Between age 3 and before questionnaire completion	180 (40.1%)
In the year preceding questionnaire completion	121 (26.9%)
After questionnaire completion	269 (59.9%)
GP diagnosis of atopic eczema	853 (32.8%)
Between age 1 and before questionnaire completion	604 (70.8%)
In the year preceding questionnaire completion	71 (8.3%)
After questionnaire completion	249 (29.2%)
GP diagnosis of allergic rhinitis	366 (14.1%)
At any time before questionnaire completion	128 (35.0%)
In the year preceding questionnaire completion	35 (9.5%)
After questionnaire completion	238 (65.0%)

*Note*: Values are mean (SD) or frequency (%).

^a^ In the year preceding questionnaire completion.

### 3.2 Concordance of parent reports  with temporal GP Condiagnoses

The agreement metrics for the primary analysis comparing parent reports to GP records from the corresponding time period are detailed in Tables 2 and 3.

**TABLE 2:** Comparison of parent‐reported vs. GP‐recorded prevalence (%, 95% CI), absolute difference, and agreement for childhood asthma, atopic eczema, and allergic rhinitis.

	Prevalence		Absolute difference	% Overall agreement	Cohen's kappa	PABAK^a^
	A: Parent‐reported	B: GP diagnosis	(A–B)			
Asthma						
Wheeze symptoms^b^	18.6 (17.0, 20.2)	5.2 (4.3, 6.1)	13.4 (11.6, 20.2)	85.2 (84.0, 86.6)	0.32 (0.29, 0.36)	0.70 (0.67, 0.73)
Severe wheeze symptoms^b^	6.8 (5.7, 7.8)		1.5 (0.2, 2.9)	93.1 (92.0, 94.1)	0.39 (0.35, 0.43)	0.86 (0.84, 0.88)
GP diagnosis of asthma^c^	8.6 (7.5, 9.7)	7.7 (6.7, 8.8)	0.8 (−0.7, 2.4)	95.3 (94.3, 96.1)	0.68 (0.64, 0.73)	0.91 (0.89, 0.92)
Atopic eczema						
Eczema symptoms^b^	16.3 (14.8, 17.8)	3.0 (2.3, 3.7)	13.3 (11.7, 15.0)	82.9 (81.3, 84.4)	0.07 (0.04, 0.10)	0.66 (0.63, 0.69)
Severe eczema symptoms^b^	2.3 (1.7, 2.9)		2.3 (1.7, 2.9)	95.2 (94.2, 96.0)	0.07 (0.03, 0.11)	0.90 (0.88, 0.92)
GP diagnosis of atopic eczema^c^	22.6 (21.0, 24.2)	25.8 (24.0, 27.5)	3.1 (0.6, 5.5)	80.0 (78.4, 81.6)	0.46 (0.42, 0.50)	0.60 (0.57, 0.63)
Allergic rhinitis						
Allergic rhinitis symptoms^b^	14.7 (13.3, 16.1)	1.5 (0.1, 0.2)	13.2 (11.7, 14.7)	84.9 (83.4, 86.4)	0.04 (0.02, 0.06)	0.70 (0.66, 0.73)
GP diagnosis of allergic rhinitis^c^	3.2 (2.5, 3.9)	5.4 (4.5, 6.3)	2.2 (1.0, 3.4)	96.6 (92.6, 94.6)	0.23 (0.19, 0.27)	0.87 (0.85, 0.89)

^a^ Prevalence‐adjusted Bias‐adjusted Kappa.

^b^ Based on a GP diagnosis recorded in the 12 months preceding questionnaire completion.

^c^ Based on a GP diagnosis recorded any time up to questionnaire completion (from age 3 for asthma; from age 1 for eczema; any age for allergic rhinitis).

**TABLE 3:** Sensitivity, specificity, positive predictive value (PPV) and negative predictive value (NPV) assessing agreement between parent‐reported symptoms and GP‐recorded diagnoses of wheeze/asthma, atopic eczema, and hay fever in the cross‐sectional analysis.

Parent‐reported outcomes	Sensitivity	Specificity	PPV	NPV
Wheeze/asthma				
Wheeze symptoms^a^	24.2 (20.3, 28.6)	99.2 (98.6, 99.15)	86.8 (79.4, 92.2)	85.1 (83.6, 86.6)
Severe wheeze symptoms^a^	37.6 (30.0, 45.7)	97.1 (96.3, 97.8)	48.8 (39.6, 58.0)	95.6 (94.6, 96.4)
GP diagnosis of asthma^b^	67.5 (60.5, 73.9)	97.9 (97.2, 98.5)	75.0 (68.0, 81.1)	97.0 (96.2, 97.7)
Atopic eczema				
Eczema symptoms^a^	7.1 (4.7, 10.1)	97.8 (97.0, 98.4)	38.0 (26.8, 50.3)	84.3 (82.8, 85.8)
Severe eczema symptoms^a^	11.1 (4.2, 22.6)	97.2 (96.4, 97.8)	8.5 (3.2, 17.5)	97.9 (97.2, 98.4)
GP diagnosis of eczema^b^	62.8 (58.5, 66.9)	85.1 (83.4, 86.7)	55.3 (51.2, 59.3)	88.6 (87.0, 90.1)
Allergic rhinitis				
Allergic rhinitis symptoms^a^	3.8 (2.0, 6.3)	98.9 (98.3, 99.3)	37.1 (21.5, 55.1)	85.7 (84.2, 87.1)
GP diagnosis of allergic rhinitis^b^	35.5 (24.9, 47.3)	95.6 (94.6, 96.4)	21.1 (14.4, 29.2)	97.8 (97.1, 98.4)

*Note*: Values are % (95% CI).

^a^ Based on a GP diagnosis recorded in the 12 months preceding questionnaire completion.

^b^ Based on a GP diagnosis recorded any time up to questionnaire completion (from age 3 for asthma; from age 1 for eczema; any age for allergic rhinitis).

#### 3.2.1 Asthma/Wheeze

Parent‐reported wheeze was more common than GP‐diagnosed asthma. Agreement for parent‐reported wheeze was moderate (Kappa = 0.32), though PABAK was substantially higher (0.70). The sensitivity for identifying a GP diagnosis was low at 24.2%. For reports of severe wheeze, the Kappa was similar (0.39), but the PABAK indicated very good agreement (0.86). Agreement for formal “ever diagnosed” report was good (Kappa = 0.68, PABAK = 0.91). This was driven by high specificity (98%) but only moderate sensitivity (68%).

#### 3.2.2 Atopic eczema

Parent reports of eczema symptoms were effective at ruling out a GP diagnosis (NPV = 84%), but agreement was poor (Kappa = 0.07). This was largely due to the prevalence imbalance in the data; after adjusting for this, the PABAK (0.66) indicated substantial agreement. For severe symptoms, the Kappa remained low at 0.07, though the PABAK was higher (0.90). Agreement for a formal “ever diagnosed” report was moderate (Kappa = 0.46, PABAK = 0.60).

#### 3.2.3 Allergic rhinitis

For recent symptoms of allergic rhinitis, agreement was poor (Kappa = 0.04), with very low sensitivity (3.8%), though the PABAK suggested higher potential agreement (0.70). Agreement for a formal “ever diagnosis” report was fair (Kappa = 0.23), but the PABAK was very good (0.87). This result was characterized by high specificity (96%), but low sensitivity (36%).

### 3.3 Concordance of parent symptom reports with cumulative GP records

The secondary analysis compared 12‐month symptom reports against the cumulative (lifetime) GP record (Tables S1 and S2). For wheeze, this extended timeframe yielded marginally better agreement for general symptoms, while agreement for severe symptoms was comparable. Sensitivity improved for both symptom types. For eczema, using the cumulative record dramatically increased the number of GP diagnoses compared to the 12‐month record. This, in turn, significantly reduced the sensitivity of parent‐reported symptoms. For allergic rhinitis, the Kappa value and sensitivity improved with the extended timeframe, but the PABAK remained similar to the primary analysis.

### 3.4 Influencing factors

Tables 4, 5, 6 present the factors associated with reporting discrepancies. For asthma, 45 parents (1.7%) underreported and 119 (4.6%) overreported. Mothers of girls were less likely to underreport, and each additional GP visit was associated with a 14% increase in the odds of underreporting. Overreporting was less likely among older mothers, those with female offspring, and those with degree‐level education. South Asian mothers and those with more frequent GP visits were more likely to overreport. For eczema, 270 (9.1%) underreported and 252 (9.7%) overreported. South Asian mothers, those with a history of eczema, and those with more frequent GP visits were more likely to underreport; however, the association with GP visits was no longer significant in the fully adjusted model. Overreporting was less likely among South Asian and other minority ethnic mothers and multiparous women. For allergic rhinitis, 101 (3.4%) underreported and 67 (2.6%) overreported. Underreporting was more common among non‐White European mothers, those with a personal diagnosis of allergic rhinitis, and those with more frequent GP visits. South Asian mothers were less likely to overreport, though more GP visits increased the odds.

**TABLE 4:** Factors associated with parent under‐ and overreporting of a GP‐diagnosis of asthma.

Characteristic	Underreport (*n* = 45; 1.7%)		Overreport (*n* = 119; 4.6%)	
	Unadjusted	Adjusted^a^	Unadjusted	Adjusted^a^
Age at delivery (years)	0.96 (0.92, 1.01)	0.97 (0.91, 1.03)	0.94 (0.91, 0.98)	0.94 (0.90, 0.98)
Ethnic group				
White European	1.00	1.00	1.00	1.00
South Asian	1.20 (0.59, 2.46)	1.08 (0.46, 2.54)	1.65 (1.05, 2.60)	1.65 (0.98, 2.68)
Other ethnic group	1.73 (0.54, 5.58)	2.26 (0.66, 7.73)	0.76 (0.26, 2.21)	0.90 (0.31, 2.59)
BMI				
Healthy weight	1.00	1.00	1.00	1.00
Overweight	1.21 (0.61, 2.39)	1.44 (0.71, 2.92)	1.08 (0.71, 1.65)	1.25 (0.82, 1.91)
Obese	1.04 (1.47, 2.31)	1.02 (0.44, 2.39)	1.00 (0.60, 1.68)	1.10 (0.65, 1.85)
Smoked during pregnancy				
No	1.00	1.00	1.00	1.00
Yes	1.73 (0.76, 3.93)	1.68 (0.60, 4.94)	0.94 (0.50, 1.78)	0.89 (0.44, 1.78)
Educational attainment				
Less than degree level	1.00	1.00	1.00	1.00
Degree level	1.17 (0.61, 2.22)	1.27 (0.60, 2.70)	0.48 (0.29, 0.79)	0.55 (0.34, 0.92)
Parity				
Nulliparous	1.00	1.00	1.00	1.00
Multiparous	0.75 (0.41, 1.39)	0.96 (0.4, 3 2.14)	0.92 (0.63, 1.35)	1.10 (0.74, 1.65)
Mother has a GP diagnosis of asthma				
No	1.00	1.00	1.00	1.00
Yes	1.90 (0.98, 3.67)	1.89 (0.92, 3.87)	2.00 (1.32, 3.03)	2.05 (1.33, 3.16)
Sex of child				
Male	1.00	1.00	1.00	1.00
Female	0.36 (0.18, 0.70)	0.33 (0.17, 0.67)	0.67 (0.46, 0.99)	0.62 (0.42, 0.90)
GP attendances by child	1.14 (1.09, 1.18)	1.14 (1.09, 1.18)	1.07 (1.04, 1.10)	1.05 (1.02, 1.09)

*Note*: Odds ratios (OR) with 95% confidence intervals (95% CI) are reported.

^a^ Adjusted for mother's age at delivery, ethnicity, BMI, smoking during pregnancy, educational attainment, parity, maternal diagnosis of each relevant condition, child sex, and frequency of GP attendances.

**TABLE 5:** Factors associated with parent under‐ and overreporting of a GP‐diagnosis of atopic eczema.

Characteristic	Underreport (*n* = 270; 9.1%)		Overreport (*n* = 252; 9.7%)	
	Unadjusted	Adjusted^a^	Unadjusted	Adjusted^a^
Age at delivery (years)	1.00 (0.97, 1.02)	1.00 (0.98, 1.03)	1.00 (0.98, 1.02)	1.01 (0.98, 1.04)
Ethnic group				
White European	1.00	1.00	1.00	1.00
South Asian	1.65 (1.22, 2.23)	1.60 (1.15, 2.22)	0.44 (0.33, 0.58)	0.43 (0.32, 0.59)
Other ethnic group	1.39 (0.78, 2.48)	1.41 (0.79, 2.53)	0.47 (0.25, 0.87)	0.47 (0.25, 0.88)
BMI				
Healthy weight	1.00	1.00	1.00	1.00
Overweight	0.81 (0.60, 1.09)	0.83 (0.61, 1.14)	1.22 (0.91, 1.65)	1.24 (0.92, 1.69)
Obese	0.89 (0.64, 1.24)	0.93 (0.66, 1.32)	0.95 (0.67, 1.36)	0.89 (0.61, 1.29)
Smoked during pregnancy				
No	1.00	1.00	1.00	1.00
Yes	0.90 (0.58, 1.39)	1.23 (0.76, 1.98)	1.40 (0.94, 2.09)	0.92 (0.60, 1.43)
Educational attainment				
Less than degree level	1.00	1.00	1.00	1.00
Degree level	1.16 (0.89, 1.53)	1.20 (0.90, 1.60)	1.04 (0.77, 1.39)	0.97 (0.71, 1.32)
Parity				
Nulliparous	1.00	1.00	1.00	1.00
Multiparous	0.92 (0.70, 1.19)	0.90 (0.67, 1.22)	0.66 (0.50, 0.85)	0.70 (0.52, 0.94)
Mother has a GP diagnosis of atopic dermatitis				
No	1.00	1.00	1.00	1.00
Yes	1.53 (1.18, 1.99)	1.47 (1.12, 1.91)	1.07 (0.80, 1.43)	1.12 (0.84, 1.50)
Sex of child				
Male	1.00	1.00	1.00	1.00
Female	0.96 (0.75, 1.23)	0.96 (0.75, 1.24)	1.00 (0.78, 1.31)	1.03 (0.79, 1.34)
Number of GP attendances by child	1.05 (1.03, 1.08)	1.04 (1.01, 1.07)	1.00 (0.97, 1.04)	1.02 (0.98, 1.06)

*Note*: Odds ratios (OR) with 95% confidence intervals (95% CI) are reported.

^a^ Adjusted for mother's age at delivery, ethnicity, BMI, smoking during pregnancy, educational attainment, parity, maternal diagnosis of each relevant condition, child sex, and frequency of GP attendances.

**TABLE 6:** Factors associated with parent under‐ and overreporting of a GP diagnosis of allergic rhinitis.

Characteristic	Underreport (*n* = 101; 3.4%)		Overreport (*n* = 67; 2.6%)	
	Unadjusted	Adjusted^a^	Unadjusted	Adjusted^a^
Age at delivery (years)	0.97 (0.94, 1.01)	0.97 (0.94, 1.02)	0.97 (0.92, 1.01)	0.97 (0.92, 1.02)
Ethnic group				
White European	1.00	1.00	1.00	1.00
South Asian	2.66 (1.52, 4.66)	2.43 (1.28, 4.62)	0.49 (0.30, 0.80)	0.43 (0.23, 0.79)
Other ethnic group	2.84 (1.18, 6.82)	2.92 (1.15, 7.42)	0.47 (0.14, 1.56)	0.50 (0.15, 1.68)
BMI				
Healthy weight	1.00	1.00	1.00	1.00
Overweight	0.79 (0.49, 1.28)	0.90 (0.55, 1.48)	0.80 (0.53, 1.49)	0.83 (0.44, 1.56)
Obese	0.88 (0.52, 1.48)	1.06 (0.61, 1.83)	1.56 (0.88, 2.76)	1.38 (0.78, 2.44)
Smoked during pregnancy				
No	1.00	1.00	1.00	1.00
Yes	0.79 (0.38, 1.65)	1.33 (0.57, 3.16)	2.08 (1.10, 3.93)	1.27 (0.61, 2.66)
Educational attainment				
Less than degree level	1.00	1.00	1.00	1.00
Degree level	1.45 (0.96, 2.21)	1.55 (0.97, 2.47)	0.68 (0.37, 1.23)	0.79 (0.43, 1.48)
Parity				
Nulliparous	1.00	1.00	1.00	1.00
Multiparous	0.73 (0.48, 1.09)	0.79 (0.49, 1.28)	0.94 (0.57, 1.57)	1.26 (0.70, 2.27)
Mother has a GP diagnosis allergic rhinitis				
No	1.00	1.00	1.00	1.00
Yes	1.76 (1.16, 2.66)	1.65 (1.09, 2.52)	1.22 (0.72, 2.07)	1.20 (0.71, 2.04)
Sex of child				
Male	1.00	1.00	1.00	1.00
Female	0.77 (0.51, 1.15)	0.77 (0.51, 1.17)	0.86 (0.53, 1.40)	0.86 (0.53, 1.42)
Number of GP attendances by child	1.10 (1.07, 1.14)	1.09 (1.05, 1.12)	1.08 (1.04, 1.12)	1.08 (1.05, 1.13)

*Note*: Odds ratios (OR) with 95% confidence intervals (95% CI) are reported.

^a^ Adjusted for mother's age at delivery, ethnicity, BMI, smoking during pregnancy, educational attainment, parity, maternal diagnosis of each relevant condition, child sex, and frequency of GP attendances.

### 3.5 Predictive validity

Parent reported symptoms and diagnoses strongly predicted a future diagnosis for all three conditions (Table 7). A parent‐reported asthma diagnosis increased the risk of a future GP diagnosis more than fivefold. For eczema, a reported diagnosis doubled the risk. For allergic rhinitis, a reported diagnosis tripled the likelihood. Reports of severe symptoms consistently showed a higher predictive value.

**TABLE 7:** Predictive validity of parent‐reported symptoms and diagnoses with a subsequent GP‐recorded diagnosis.

Parent reported symptom/diagnosis	*N*	Unadjusted RR (95% CI)	Fully adjusted^a^ RR (95% CI)
Asthma	2414^b^		
Wheeze	390^c^	3.21 (2.69, 3.84)	2.56 (2.10, 3.11)
Severe wheeze	115^c^	5.13 (3.60, 7.29)	4.09 (2.82, 5.93)
GP diagnosis of asthma	119^c^	6.62 (4.69, 9.35)	5.14 (3.47, 7.61)
Atopic eczema	1990^b^		
Symptoms	178^c^	1.96 (1.41, 2.72)	1.96 (1.42, 2.70)
Severe symptoms	15^c^	4.66 (1.67, 12.99)	4.37 (1.74, 10.98)
GP diagnosis of atopic eczema	252^c^	2.16 (1.54, 3.02)	1.99 (1.52, 2.60)
Allergic rhinitis	2446^b^		
Symptoms	366^c^	2.02 (1.60, 2.55)	1.91 (1.50, 2.43)
GP diagnosis of allergic rhinitis	67^c^	3.44 (2.04, 5.80)	3.36 (1.98, 5.72)

*Note*: Relative risks (RR) with 95% confidence intervals (95% CI) CI are presented for unadjusted and adjusted analysis on imputed datasets.

^a^ Adjusted for mother's age at delivery, ethnicity, BMI, smoking during pregnancy, educational attainment, parity, maternal diagnosis of each relevant condition, child sex, and frequency of GP attendances.

^b^ Denominator (diagnoses already documented in primary care records before parental report were excluded).

^c^ Number of symptoms or diagnoses reported by parents prior to a corresponding diagnosis in primary care records.

## 4 DISCUSSION

This study revealed variable agreement between parent‐reported allergic conditions and GP‐recorded diagnoses. For asthma and allergic rhinitis, Kappa scores were low to moderate, but high PABAK values suggest these discrepancies were driven more by the low prevalence of recorded diagnoses and differing clinical thresholds rather than fundamental disagreement. In contrast, for eczema, moderate PABAK values suggest more genuine variations in how parents and GPs interpret the condition. For all conditions, parent reports were highly reliable for ruling out a diagnosis (high specificity and NPV).

Several demographic and clinical factors influenced reporting accuracy. Notably, South Asian ethnicity and higher frequency of GP visits were associated with both under‐ and overreporting depending on the condition, suggesting these factors are complex signals that warrant further investigation.

Despite discrepancies in agreement, parent reports at age 4–5 demonstrate strong predictive validity. An “ever” diagnosis report from a parent significantly increased the risk of a future GP diagnosis more than fivefold for asthma, threefold for allergic rhinitis, and twofold for eczema, with reports of severe symptoms showing the highest predictive value.

### 4.1 Comparison with other studies

Our findings align with previous research, which consistently shows discrepancies between parent reports and GP diagnoses for conditions like asthma and eczema. For instance, parent‐reported wheezing frequently exceeds GP‐diagnosed asthma rates, a common finding where prevalence estimates vary depending on the diagnostic definition, the child's age, and who is reporting.^7^, ^9^, ^19^ It is common for a child to receive an “asthma” label from one source, such as a parent, but not have it recorded in their medical records.^20^ This discrepancy tends to diminish as children get older, potentially due to the resolution of temporary, non‐asthmatic wheezing or improved parental symptom recognition.^9^ However, accuracy also depends on disease onset timing, with parents being more reliable for early‐onset asthma.^21^ Similar inconsistencies exist for atopic dermatitis, where agreement can sometimes decrease with age.^11^ A key factor influencing agreement across all conditions is symptom severity. Concordance between parents and doctors is consistently higher for more severe cases^9^, ^11^, ^22^ and more objective events like recent medication use.^19^ Despite the variability, our analysis confirms that parental reports are exceptionally valuable for ruling out conditions, as demonstrated by their high specificity and NPV, which aligns with other research.^9^, ^22^ This is strongly supported by studies that have found that a parent's denial of their child's asthma is highly trustworthy.^21^

### 4.2 Reasons for disagreement

Discrepancies may stem from several factors. A primary methodological reason is the potential for a mismatch between the timeframe of the parent‐reported outcomes and the GP diagnosis, which can decrease agreement and affect predictive ability. Our secondary analysis, which compared symptoms “in the last 12 months” against a child's cumulative medical history, highlighted this temporal mismatch. For instance, a parent who accurately reports their child has been free of eczema for the past year would appear to “disagree” with a GP record of eczema in early childhood. Furthermore, our study design itself can create apparent disagreement due to the age‐based criteria applied to the GP records. We only considered GP diagnoses of asthma from age three and eczema from age one to avoid misclassifying transient infant symptoms. However, a parent may accurately recall a definitive diagnosis of eczema made at 6 months of age. Because this diagnosis would be excluded by our study's definition, the parent's correct report would be incorrectly classified as a disagreement, artificially lowering the measured agreement and sensitivity. This effect, however, is a direct consequence of our a priori case definition, which was implemented to increase the clinical specificity of the outcomes. The age criteria were essential for distinguishing persistent disease from more transient infantile symptoms, such as early‐life wheezing. Therefore, while this methodological choice reduces agreement on diagnoses made in infancy, it strengthens our confidence that the results for the included cases are clinically valid and meaningful. Other factors include challenges with recall for “ever” diagnosis and differing perspectives. A parent might, for example, remember informal advice that was never formally coded. Furthermore, inconsistencies can arise from differing perspectives and terminology; parents observe symptoms within the context of everyday life and interpret them subjectively, while GPs rely on structured clinical assessments. Crucially, even for recent symptoms, a diagnosis can only be recorded if a GP is consulted. A parent may accurately report a symptom from the last 12 months that is not reflected in the GP record for several valid reasons. They may have sought advice elsewhere, such as an emergency department, or managed the condition with over‐the‐counter treatments. Additionally, parents may not feel a GP consultation is necessary if they are experienced in managing the symptoms based on familiarity with the condition in siblings or themselves. These scenarios lead to a genuine symptoms experience not being captured in the primary care record, which influences the interpretation of our agreement and predictive metrics. Finally, a parents' medical understanding and cultural background shape their health‐seeking behaviors, and even when a child does see a GP, their symptoms may go undocumented if they are not the main reason for the visit.

### 4.3 Factors influencing underreporting and overreporting

Parental reporting accuracy is shaped by demographic and behavioral factors. Discrepancies linked to ethnicity and education underscore concerns about health inequalities. Frequent GP consultations were associated with both under‐ and overreporting, perhaps reflecting two distinct parental groups: some with heightened health anxiety leading to overreporting, and others whose frequent visits for unrelated issues lead to atopic conditions being underrecorded. However, the effect sizes were generally modest, so these findings should be considered hypothesis‐generating, highlighting consistent signals like ethnicity and healthcare use that warrant further investigation.

### 4.4 Predictive ability

Parent‐reported symptoms and diagnoses demonstrated strong predictive validity, likely reflecting parents' early recognition of key symptoms, informed by experience or family history. In conditions like asthma and eczema with common diagnostic delays, early parental reporting can capture emerging symptoms not yet clinically recorded. This underscores the importance of integrating parent perspectives into clinical care and research.

### 4.5 Implications for epidemiological research

The use of routine EHR data must be viewed within the shift away from survey‐based epidemiology. While surveys offer valuable insights, they face challenges like high costs and recall bias. EHRs provide a scalable alternative but may miss milder cases and lack subjective perspectives. Understanding the mismatches between parent reports and GP diagnoses is essential for interpreting EHR‐based research. Despite current gaps, EHRs are becoming more comprehensive,^23^ and advances like Natural Language Processing (NLP) are improving data extraction accuracy, even from unstructured clinical notes,^24^ enhancing their utility for population health research.

### 4.6 Strengths and limitations

The BiB study provides a robust platform for examining diagnostic accuracy, with its large, population‐based design and strong representation of South Asian families—an often‐underrepresented group in health research. Its longitudinal design enables the tracking of parent‐reported symptoms and subsequent GP diagnoses over time. Linkage to EHRs provides objective clinical data, reducing recall bias and allowing for more reliable comparisons. Additionally, the inclusion of rich socioeconomic data supports a broader understanding of the factors influencing symptom recognition and reporting.

However, the study has limitations. We defined cases using GP‐recorded diagnostic codes, which represent the outcome of routine clinical care. We acknowledge that these diagnoses are not always supported by objective tests and therefore do not represent a perfect ‘gold standard’. However, as our aim was to assess the concordance between parental reports and the formally documented diagnosis that dictates clinical management, we consider the GP record to be the most relevant real‐world benchmark for this study. Our analysis used only structured EHR data, excluding narrative notes that might contain diagnostic details. Additionally, the data are drawn from approximately 80 GP practices, and we could not account for potential clustering effects or heterogeneity in diagnostic and recording practices at the clinician or practice level, which may contribute to the disagreement observed. Furthermore, our reliance on EHRs means we cannot distinguish between genuinely disease‐free children and those with undiagnosed conditions, an inherent limitation of routine clinical data. Methodologically, survival analysis would have provided a more precise model of time‐to‐diagnosis, but small numbers of post‐questionnaire diagnoses limited its feasibility. As a result, Poisson regression was used, trading temporal precision for statistical power. Attempts to model agreement as a multinomial outcome encompassing agreement, under‐reporting, and over‐reporting were also constrained by small subgroup sizes, which limits statistical power and generalizability. The wide confidence intervals around some estimates reflect uncertainty, and the exploratory findings on factors influencing reporting should not be over‐interpreted.

## 5 CONCLUSIONS

In conclusion, our research demonstrates that neither parent‐reported data nor electronic health records (EHRs) alone provide a complete picture of childhood allergic conditions. While parental reports capture valuable information on symptoms and predictive insights, they are subject to reporting biases. Conversely, EHRs provide objective diagnostic data but may miss unreported or milder cases. Therefore, we recommend an integrated approach for epidemiological research, combining the rich, subjective experience from parent reports with the objective, longitudinal data in EHRs. This synergy allows for a more comprehensive understanding of disease burden, helps identify unmet clinical needs, and ensures that the essential patient voice is not lost but enriched by the increasing reliance on routine data.

## AUTHOR CONTRIBUTIONS

**Gillian Santorelli:** Conceptualization; writing – original draft; methodology; formal analysis; project administration; data curation. **Lucy Pembrey:** Writing – review and editing. **John Wright:** Funding acquisition; writing – review and editing.

## FUNDING INFORMATION

This work was supported by a joint grant from the UK Medical Research Council (MRC) and UK Economic and Social Science Research Council (ESRC) (MR/N024391/1); the British Heart Foundation (CS/16/4/32482); a Wellcome Trust Infrastructure Grant (WT101597MA); the National Institute for Health Research under its Applied Research Collaboration for Yorkshire and Humber (NIHR200166) and UK National Institute for Health and Care Research (NIHR) Health Technology Assessment Programme (16/150/06). The National Institute for Health Research Clinical Research Network provided research delivery support for this study. The views expressed in this publication are those of the authors and not necessarily those of the National Institute for Health Research or the Department of Health and Social Care. The MeDALL collaboration is funded by European Union Seventh Framework Programme under grant agreement number 261357.

## CONFLICT OF INTEREST STATEMENT

None declared.

## PEER REVIEW

The peer review history for this article is available at https://www.webofscience.com/api/gateway/wos/peer‐review/10.1111/pai.70166.

## Supporting information


Table S1.

## References

[1] Matterne U , Schmitt J , Diepgen L , Apfelbacher C . Children and adolescents' health‐related quality of life in relation to eczema, asthma and hay fever: results from a population‐based cross‐sectional study. Qual Life Res. 2011;20:1295‐1305. 21347571 10.1007/s11136-011-9868-9

[2] Dawson A . The costs of atopic dermatitis. 2023. https://demos.co.uk/research/the‐costs‐of‐atopic‐dermatitis/

[3] Allergy UK . https://www.allergyuk.org/.

[4] Mukherjee M , Stoddart A , Gupta RP , et al. The epidemiology, healthcare and societal burden and costs of asthma in the UK and its member nations: analyses of standalone and linked national databases. BMC Med. 2016;14:113. 27568881 10.1186/s12916-016-0657-8 PMC5002970

[5] Asthma + Lung UK . Asthma Facts and Stats. https://www.asthmaandlung.org.uk/

[6] Lemonnier N , Melén E , Jiang Y , et al. A novel whole blood gene expression signature for asthma, dermatitis, and rhinitis multimorbidity in children and adolescents. Allergy. 2020;75:3248‐3260. 32277847 10.1111/all.14314 PMC9302020

[7] Eijkemans M , Mommers M , Thijs C . Comparison of parent reported physician diagnosed asthma and general practitioner registration. J Asthma. 2023;60:673‐681. 35686624 10.1080/02770903.2022.2087189

[8] Cornish RP , Henderson J , Boyd AW , Granell R , van Staa T , Macleod J . Validating childhood asthma in an epidemiological study using linked electronic patient records. BMJ Open. 2014;4:e005345. 10.1136/bmjopen-2014-005345 PMC4010849 24760357

[9] Griffiths LJ , Lyons RA , Bandyopadhyay A , et al. Childhood asthma prevalence: cross‐sectional record linkage study comparing parent‐reported wheeze with general practitioner‐recorded asthma diagnoses from primary care electronic health records in Wales. BMJ Open Respir Res. 2018;5:e000260. 10.1136/bmjresp-2017-000260 PMC5759709 29333271

[10] Matthewman J , Mulick A , Dand N , et al. Disagreement concerning atopic dermatitis subtypes between an English prospective cohort (ALSPAC) and linked electronic health records. Clin Exp Dermatol. 2024;49:1537‐1546. 38751343 10.1093/ced/llae196 PMC11583923

[11] Peng Z , Braig S , Kurz D , et al. Trajectory and determinants of agreement between parental and physicians' reports of childhood atopic dermatitis. Pediatr Allergy Immunol. 2022;33:e13855. 36156820 10.1111/pai.13855

[12] McEachan RRC , Santorelli G , Watmuff A , et al. Cohort profile update: Born in Bradford. Int J Epidemiol. 2024;53:dyae037. 38552669 10.1093/ije/dyae037 PMC11065350

[13] Wright J , Small N , Raynor P , et al. Cohort profile: the born in Bradford multi‐ethnic family cohort study. Int J Epidemiol. 2013;42:978‐991. 23064411 10.1093/ije/dys112

[14] Bousquet J , Anto J , Auffray C , et al. MeDALL (mechanisms of the development of ALLergy): an integrated approach from phenotypes to systems medicine. Allergy. 2011;66:596‐604. 21261657 10.1111/j.1398-9995.2010.02534.x

[15] Hohmann C , Pinart M , Tischer C , et al. The development of the MeDALL core questionnaires for a harmonized follow‐up assessment of eleven European birth cohorts on asthma and allergies. Int Arch Allergy Immunol. 2014;163:215‐224. 24642608 10.1159/000357732

[16] Souza da Cunha S , Santorelli G , Pembrey L . Defining cases of asthma, eczema and allergic rhinitis using electronic health records in the born in Bradford birth cohort. Clin Exp Allergy. 2023;53:664‐667. 36756903 10.1111/cea.14291 PMC10946775

[17] Landis JKG . The measurement of observer agreement for categorical data. Biometrics. 1977;33:159‐174. 843571

[18] Byrt T , Bishop J , Carlin JB . Bias, prevalence and kappa. J Clin Epidemiol. 1993;46:423‐429. 8501467 10.1016/0895-4356(93)90018-v

[19] Korsten K , Naaktgeboren CA , Bont LJ , van der Ent CK , de Hoog MLA . Defining asthma in children: how well do parents, doctors and spirometry agree? ERJ Open Res. 2020;6:00348‐2019. 33043055 10.1183/23120541.00348-2019 PMC7533381

[20] Canova C , Harris JM , Mills P , et al. Epidemiological measures of childhood asthma: cross‐sectional and longitudinal consistency. Respir Med. 2012;106:1226‐1235. 22705292 10.1016/j.rmed.2012.05.008

[21] Kuiper IN , Svanes C , Benediktsdottir B , et al. Agreement in reporting of asthma by parents or offspring – the RHINESSA generation study. BMC Pulm Med. 2018;18:122. 30053806 10.1186/s12890-018-0687-4 PMC6062946

[22] Peltonen H , Kukkonen AK , Korkalo L , et al. Associations of multiple lifestyle behaviors with allergic disease symptoms and sensitization in 9–11‐year‐old Finnish children. BMC Pediatr. 2024;24:749. 39558290 10.1186/s12887-024-05218-8 PMC11575105

[23] Shen Y , Yu J , Zhou J , Hu G . Twenty‐five years of evolution and hurdles in electronic health records and interoperability in medical research: comprehensive review. J Med Internet Res. 2025;27:e59024. 39787599 10.2196/59024 PMC11757985

[24] Sim J‐A , Huang X , Horan MR , et al. Natural language processing with machine learning methods to analyze unstructured patient‐reported outcomes derived from electronic health records: a systematic review. Artif Intell Med. 2023;146:102701. 38042599 10.1016/j.artmed.2023.102701 PMC10693655

