# Supplementary material for: Agreement between parent reported and clinical coding of asthma, eczema and allergic rhinitis: The multi‐ethnic Born in Bradford cohort
Source: Pediatr Allergy Immunol. 2025 Aug 7;36(8):e70166. doi: 10.1111/pai.70166 (PMC12329701; doi:10.1111/pai.70166)
Supplement: Supplementary file 1 — Table S1. [file PAI-36-e70166-s001.docx]

Supplementary Table 1: Comparison of parent-reported vs. GP-recorded prevalence (%, 95% CI), absolute difference and agreement for childhood asthma, atopic eczema, and allergic rhinitis using cumulative GP records.

|  | **Prevalence** | | **Absolute difference**  **(A – B)** | **% Overall agreement** | **Cohen’s kappa** |  |
| --- | --- | --- | --- | --- | --- | --- |
|  | **A: Parent-reported** | **B: GP diagnosis†** |  |  |  | **PABAK^ǂ^** |
| Wheeze symptoms | 18.6 (17.0, 20.2) | 7.7 (6.7, 8.8) | 10.9 (9.0, 12.8) | 87.0 (85.5, 88.0) | 0.44 (0.41, 0.48) | 0.74 (0.71, 0.77) |
| Severe wheeze symptoms | 6.8 (5.7, 7.8) |  | 1.0 (0.5, 2.4) | 93.0 (91.9, 94.0) | 0.48 (0.44, 0.52) | 0.86 (0.84, 0.88) |
|  |  |  |  |  |  |  |
| Eczema symptoms | 16.3 (14.8, 17.8) | 25.8 (24.0, 27.5) | 9.4 (7.1, 11.7) | 78.7 (77.0, 80.3) | 0.37 (0.23, 0.41) | 0.57 (0.54, 0.61) |
| Severe eczema symptoms | 2.3 (1.7, 2.9) |  | 23.5 (21.6, 25.3) | 75.8 (74.0, 77.5) | 0.10 (0.08, 0.12) | 0.52 (0.48, 0.55) |
|  |  |  |  |  |  |  |
| Allergic rhinitis symptoms | 14.7 (13.3, 16.1) | 5.4 (4.5, 6.3) | 9.3 (4.5, 6.3) | 83.7 (82.1, 85.2) | 0.12 (0.09, 0.16) | 0.67 (0.64, 0.70) |

†A GP diagnosis recorded at any time up to the completion of the questionnaire, starting from age 3 for asthma, age 1 for eczema, or at any age for allergic rhinitis;

^ǂ^Prevalence-adjusted Bias-adjusted Kappa

Supplementary Table 2: Sensitivity, specificity, positive predictive value (PPV) and negative predictive value (NPV) assessing agreement between parent-reported symptoms and GP-recorded diagnoses† of wheeze/asthma, atopic eczema, and hay fever in the cross-sectional analysis. Values are % (95% CI).

| **Parent-reported outcomes** | **Sensitivity** | **Specificity** | **PPV** | **NPV** |
| --- | --- | --- | --- | --- |
| Wheeze symptoms | 35.8 (31.3, 40.5) | 98.7 (98.1, 99.1) | 86.1 (80.2, 90.8) | 87.0 (85.5, 88.4) |
| Severe wheeze symptoms | 55.4 (47.3, 63.3) | 95.7 (94.8, 96.5) | 48.3 (40.8, 55.9) | 96.7 (95.9, 97.4) |
| Eczema symptoms | 63.7 (58.7, 68.5) | 81.7 (79.9, 83.3) | 40.4 (36.5, 44.4) | 92.0 (90.6, 93.2) |
| Severe eczema symptoms | 83.3 (70.7, 92.1) | 75.6 (73.8, 77.3) | 7.5 (5.5, 9.8) | 99.5 (99.0, 99.8) |
| Allergic rhinitis symptoms | 13.0 (9.7, 17.0) | 95.9 (94.9, 96.7) | 35.2 (26.9, 44.1) | 86.5 (85.0, 87.9) |

†A GP diagnosis recorded at any time up to the completion of the questionnaire, starting from age 3 for asthma, age 1 for eczema, or at any age for allergic rhinitis.
